# isolateR: an R package for generating microbial libraries from Sanger sequencing data

**DOI:** 10.1093/bioinformatics/btae448

**Published:** 2024-07-11

**Authors:** Brendan Daisley, Sarah J Vancuren, Dylan J L Brettingham, Jacob Wilde, Simone Renwick, Christine V Macpherson, David A Good, Alexander J Botschner, Sandi Yen, Janet E Hill, Matthew T Sorbara, Emma Allen-Vercoe

**Affiliations:** Department of Molecular and Cellular Biology, University of Guelph, Guelph, ON N1G 2W1, Canada; Department of Molecular and Cellular Biology, University of Guelph, Guelph, ON N1G 2W1, Canada; Department of Molecular and Cellular Biology, University of Guelph, Guelph, ON N1G 2W1, Canada; Department of Molecular and Cellular Biology, University of Guelph, Guelph, ON N1G 2W1, Canada; Department of Pediatrics, School of Medicine, University of California, San Diego, United States; Larsson-Rosenquist Foundation Mother-Milk-Infant Center of Research Excellence (MOMI CORE), The Human Milk Institute (HMI), University of California, San Diego, CA 92093, United States; Department of Molecular and Cellular Biology, University of Guelph, Guelph, ON N1G 2W1, Canada; Department of Molecular and Cellular Biology, University of Guelph, Guelph, ON N1G 2W1, Canada; Department of Molecular and Cellular Biology, University of Guelph, Guelph, ON N1G 2W1, Canada; Kennedy Institute of Rheumatology, Medical Sciences Division, University of Oxford, Oxford OX1 2JD, United Kingdom; Department of Veterinary Microbiology, University of Saskatchewan, Saskatoon, SK S7N 5B4, Canada; Department of Molecular and Cellular Biology, University of Guelph, Guelph, ON N1G 2W1, Canada; Department of Molecular and Cellular Biology, University of Guelph, Guelph, ON N1G 2W1, Canada

## Abstract

**Motivation:**

Sanger sequencing of taxonomic marker genes (e.g. 16S/18S/ITS/*rpoB*/*cpn60*) represents the leading method for identifying a wide range of microorganisms including bacteria, archaea, and fungi. However, the manual processing of sequence data and limitations associated with conventional BLAST searches impede the efficient generation of strain libraries essential for cataloging microbial diversity and discovering novel species.

**Results:**

isolateR addresses these challenges by implementing a standardized and scalable three-step pipeline that includes: (1) automated batch processing of Sanger sequence files, (2) taxonomic classification *via* global alignment to type strain databases in accordance with the latest international nomenclature standards, and (3) straightforward creation of strain libraries and handling of clonal isolates, with the ability to set customizable sequence dereplication thresholds and combine data from multiple sequencing runs into a single library. The tool’s user-friendly design also features interactive HTML outputs that simplify data exploration and analysis. Additionally, *in silico* benchmarking done on two comprehensive human gut genome catalogues (IMGG and Hadza hunter-gather populations) showcase the proficiency of isolateR in uncovering and cataloging the nuanced spectrum of microbial diversity, advocating for a more targeted and granular exploration within individual hosts to achieve the highest strain-level resolution possible when generating culture collections.

**Availability and implementation:**

isolateR is available at: https://github.com/bdaisley/isolateR.

## Graphical abstract

**Figure:**
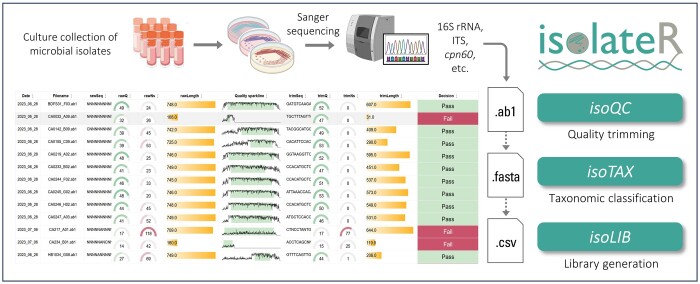

## 1 Introduction

The rapid expansion of culturomics in microbial research has emphasized the need for standardized generation of comprehensive strain libraries to catalogue the extensive variety of microorganisms identified within different environments. A single culture-based investigation can typically yield over a thousand different microbial isolates (Lagier *et al.* 2016), underscoring the challenge of processing and analyzing the vast amount of associated sequencing data. As the gold standard in microbial genomics, Sanger sequencing (Sanger *et al.* 1977) produces high-accuracy sequence data stored in ABIF formatted trace files. Several software packages including Poly Peak Parser (Hill *et al.* 2014), sangeranalyseR (Chao *et al.* 2021), and TraceTrack (Brazdilova *et al.* 2023) have recently been developed to improve aspects of analysis such as basecalling, visualization of chromatograms, and batch processing of data. Despite these advancements, there is a notable lack of software available to organize integrated taxonomic classification of Sanger sequence data or the standardized generation of strain libraries, both of which are crucial aspects limiting current microbial isolation workflows.

The 16S rRNA gene is the most commonly used marker for the taxonomic classification of cultured and uncultured bacteria and archaea (Janda and Abbott 2007). However, other marker genes such as *rpoB* (Case *et al.* 2007) and *cpn60* (Vancuren and Hill 2019) have emerged as valuable tools for their ability to delineate taxonomy with greater resolution. Additionally, for fungal isolation and classification, 18S rRNA and Internal Transcribed Spacer (ITS) regions serve as critical taxonomic markers, offering insights into fungal diversity and phylogeny (Schoch *et al.* 2012).

In terms of assigning taxonomy to marker sequence data, conventional methods typically rely on the Basic Local Alignment Search Tool (BLAST) and GenBank non-redundant nucleotide databases which contain sequences from non-type strain material (Altschul *et al.* 1997). While this approach generally offers a satisfactory estimate of taxonomy, relying on comparisons to non-type strain material, such as uncultured clone sequences, can yield high-confidence matches that overlook sequences indicative of novel species. Moreover, local alignment algorithms (e.g. the seed-and-extend heuristic used in BLAST) have limitations in accurately classifying distantly-related sequences (Tindall *et al.* 2010). Global alignment algorithms typically provide better phylogenetic discrimination. For example, clear taxonomic boundaries have been determined for bacteria and archaea based on global alignment of 16S rRNA gene sequence similarity at the species (98.7%), genus (94.5%), family (86.5%), order (82.0%), class (78.5%), and phylum (75.0%) levels (Yarza *et al.* 2014). Similar hierarchical approaches have been applied in developing rational thresholds for delimiting filamentous fungi based on ITS sequence similarity (Vu *et al.* 2019), as well as the “species hypothesis” system used in the UNITE database (Nilsson *et al.* 2019). While sequence identity thresholds are not universally applicable to all groups of taxa nor all marker sequence regions (Edgar 2018), they serve as a useful general indicator for screening of potentially novel taxa in culture collections.

There is a pressing need for an automated system to handle large volumes of Sanger sequencing data efficiently and accurately. Such a system should not only streamline sequence quality control and trimming but also incorporate a robust framework for up-to-date taxonomic assignment. Traditional methodologies fall short in this aspect, especially given the dynamic nature of microbial taxonomy and the necessity for ongoing updates against authoritative resources such as the List of Prokaryotic names with Standing in Nomenclature (LPSN) (Parte 2014).

**Figure 1.**
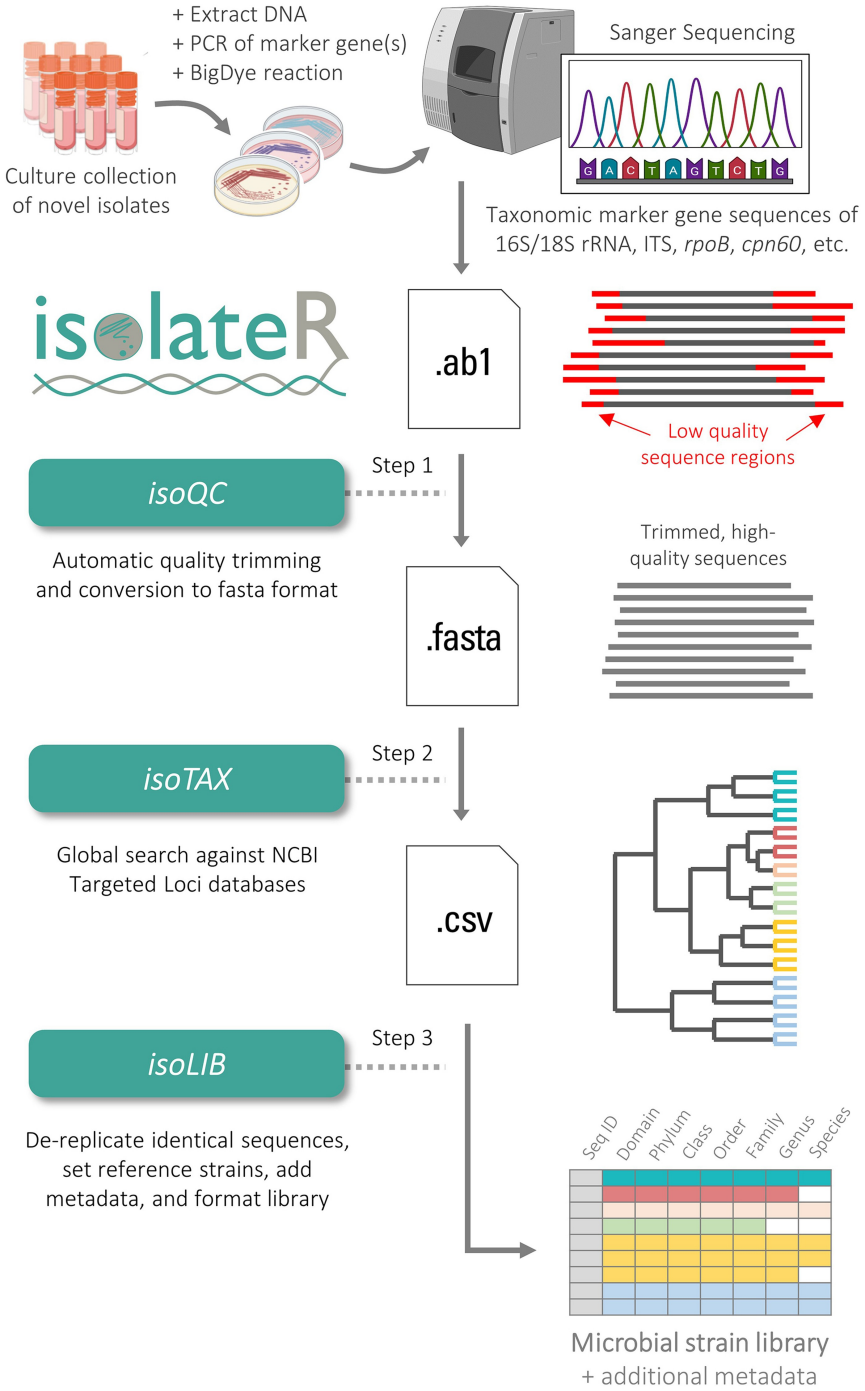
Overview of the isolateR command pipeline for microbial isolation workflows. The first step of the pipeline, *isoQC*, requires the input of taxonomic marker gene sequence trace files in ABIF format. The input sequences are automatically trimmed to remove poor quality reads (red sections) and then are categorized into PASS/FAIL groupings and converted to FASTA format for further inspection. The second step, *isoTAX*, performs taxonomic classification of the sequences by matching them against a type strain reference database for the marker gene of interest. Finally, the third step, *isoLIB*, determines the pairwise identity of sequences between one another to allow for standardized grouping of microbial isolates based on a desired similarity threshold. Ultimately, a microbial strain library is generated based on the dereplicated sequence representatives and then an interactive HTML table is output in a human-readable format. Information on the dereplicated isolates is retained. Quality metrics and other metadata about the sequences (including the dereplicated sequences) are retained and accessible through the menu buttons. Additional isolation runs can be added to the same strain library if desired and users can visualize phylogenetic relationships using several built-in functions of isolateR.

In response, we have developed a dedicated R package, isolateR, to meet the high-throughput demands of modern microbial isolation workflows (Fig. 1). This tool not only automates the quality processing of ABIF files but also integrates a global alignment method to ensure accurate taxonomic classification based on type strain reference databases, as well as aids in the organized collection of microbial sequence data. By enhancing the precision and throughput of cataloging microbial strains, isolateR enables a more comprehensive and accurate exploration of microbial diversity, paving the way for new discoveries and applications in various scientific fields.

## 2 Materials and methods

### 2.1 Software description

isolateR is designed to run entirely within an R environment from either the command line interface or through the command console of RStudio. It is compatible with all major operating systems, including Windows, macOS, and Linux. The package is efficiently designed to offer a user-friendly experience with minimal dependencies and quick installation. Detailed documentation and usage examples are available on the package’s GitHub repository (https://github.com/bdaisley/isolateR). On a standard laptop computer (8 processors @ 1.60 GHz), isolateR can process 100 Sanger sequencing files, including taxonomic annotation and strain library creation in less than 10 min using under 8 GB of RAM.

### 2.2 Data import and Sanger sequence quality processing

The first stage of analysis in the pipeline is performed *via* the “*isoQC*” function, which facilitates the batch input and processing of Sanger sequencing-derived ABIF files (.ab1 extension). Briefly, the DNA sequence trace information from each file is extracted and then the sangeranalyseR package is wrapped to perform basecalling with a signal ratio cutoff as previously described (Chao *et al.* 2021). The default value (0.33) ensures only signals greater than 1/3 of the maximum signal ratio are considered and that weaker signals are labeled as “N” (i.e. ambiguous). Subsequently, the per-base Phred quality scores are extracted and then sequences are trimmed using an adaptive approach based on the sliding window algorithm implemented in Trimmomatic (Bolger *et al.* 2014). By default, the sliding window size is set to 15 bases, whereas the quality score cutoff is set to 2/3 of the maximum Phred score per sequence.

Users can adjust the parameters for both the basecalling and trimming steps to allow removal of low-quality data which may interfere with downstream taxonomic classification and library creation steps. In cases of paired sequencing, consensus sequences from forward and reverse read pairs can be automatically generated *via* error-tolerant overlapping pattern recognition using the “sanger_consensus” function. Contig assembly from multiple (greater than two) sequence pairs is also supported *via* regex-friendly file grouping features and automatic detection of sequence directionality and overlap.

Sequences and their relevant metadata are then automatically output as an isoQC-class S4 object with slots containing the pre- and post-trimming sequence length, average quality scores, total number of N’s, and other metadata. A decision slot (pass/fail) is generated based on the final sequence quality characteristics; by default, those with a trimmed length of less than 200 bp and/or a mean Phred score of less than 20 are considered unacceptable quality. Final exports from this step include: (1) sequence files in FASTA format, (2) sequence metadata in CSV format, and (3) an interactive HTML table with descriptive statistics allowing inspection and fine tuning prior to moving to the next stage in the pipeline.

### 2.3 Taxonomic classification

The second stage of analysis is performed via the “*isoTAX*” function, which facilitates taxonomic classification based on pairwise sequence alignment and calculation of sequence similarity to the closest species representative from type strain material. This is achieved using an optimal global alignment approach, specifically by wrapping the Needleman–Wunsch algorithm implemented in VSEARCH (Rognes *et al.* 2016), in which pairwise similarity is defined as the total number of matching nucleotides in query and target sequences, excluding terminal gaps. By default, query sequences are assumed to represent a fragment of the 16S rRNA gene (i.e. the most common marker gene used for identifying bacterial or archaeal isolates) and are automatically matched against the latest version of the respective NCBI RefSeq Targeted Loci database, containing 16S rRNA sequences from bacteria and archaea type material only.

As an additional step to ensure compliance with international nomenclature standards, assigned genus and species names can also be screened against the LPSN database (Meier-Kolthoff *et al.* 2022), and automatically corrected if necessary.

Users can adjust several parameters to allow searching against other RefSeq Targeted Loci databases (e.g. ITS or 18S rRNA for identifying fungal isolates), as well as custom databases depending on the specific needs of an isolation workflow. In cases where a query sequence ambiguously matches with identical pairwise similarities to two or more database sequences representing different taxa, a decision is made to concatenate the species name and further inspection of the results are recommended. A list of closest matching species based on sequence identity are then output as an isoTAX-class S4 object with slots containing taxonomic information as well as other sequence metadata from the prior step (e.g. length, Phred quality, N’s). Higher rank taxonomic information (phylum, class, order, and family) for each sequence is further retrieved using the NCBI Entrez batch search system (Winter 2017), and a unique column is designated for each rank from phylum to species level. Subsequently, the lowest common ancestor (LCA) as approximated using established taxonomic identity thresholds (Yarza *et al.* 2014, Vu *et al.* 2019) for each sequence is highlighted to aid in the identification of potentially novel taxa. The final taxonomic results table is exported as an interactive HTML object to allow for visual inspection prior to strain library creation.

### 2.4 Generating strain libraries

The third stage of analysis is performed *via* the “*isoLIB*” function, which facilitates the de-duplication of clonal or closely related isolates based on either their closest matching database hit (method = “closest_species”) or agglomerative hierarchical-based clustering (Zou *et al.* 2020) of pairwise sequence similarities (method = “dark_mode”). The latter method is particularly effective for cataloging groups of novel microbes, i.e. dark matter, which may have close relatives within a given ecosystem or culture collection but otherwise lack reliable database sequence representation needed for accurate classification (Dueholm *et al.* 2020, 16). The default threshold value (0.995) uses a greedy incremental algorithm to iteratively assign sequences to different groups of which centroid sequence representatives have less than 99.5% pairwise similarity to one another (Rognes *et al.* 2016). Based on using 16S rRNA as a marker gene, this default threshold is expected to allow capture of intraspecies strain diversity in most communities since 98.7% pairwise similarity is the approximate cutoff for species demarcation (Kim *et al.* 2014). Alternatively, researchers seeking to develop a strain library with broad taxonomic coverage at the genus-to-species rank without regard for strain-level diversity may consider a threshold cutoff between 94.5% and 98.7% sequence similarity (Yarza *et al.* 2014). Ultimately, the generated strain library containing sequence information, metadata, and group designations is exported in table format as an interactive HTML object allowing visual inspection. If desired, the dataset can be further manually edited in place and saved as a CSV file. Users can also adjust parameters to specify a previously generated strain library. In this case, the new and old strain libraries are integrated, with priority given to the original strain groupings and designation of centroid sequence representatives in the older library. This process can be repeated as many times as necessary to support long-term and dynamic isolation workflows.

### 2.5 *In silico* benchmark analysis

To practically evaluate the *isoLIB* function in generating isolate libraries from human gut samples, we performed a simulated analysis on 6729 high-quality (HQ) genomes from the Inner Mongolian Gut Genome (IMGG) catalogue derived from fecal samples of *n* = 60 healthy adults (Jin *et al.* 2023). Briefly, *in silico* PCR was performed to extract genes of interest from each of the genomes (available here: https://figshare.com/articles/dataset/High_quality_NHMAG/19661523) using USEARCH’s “search_pcr” command (Edgar et al. 2010) with three standard bacterial-identification primer sets targeting the full-length 16S rRNA gene (Waechter *et al.* 2023), V3–V6 regions of the 16S rRNA gene (Gloor *et al.* 2010), and the universal target (UT) of the *cpn60* gene (Vancuren and Hill 2019).

A total of 6533 genomes harboring all targets were further analyzed. In cases of intragenomic heterogeneity where a genome harbored multiple marker genes with different sequences, the consensus was determined using the DECIPHER package (Wright 2016) (“ConsensusSequence” command, ignoreNonBases = TRUE) to derive the most likely single sequence variant expected from Sanger sequencing-based identification during isolation workflows. Subsequently, each of the genomes (representative of distinct isolates in this simulation) were iteratively grouped based on marker gene sequence similarities of between 85% and 100% using isoLIB (“group_cutoff” = 0.85–1). Pooled library generation included all 6533 genomes whereas the individual library generations included genomes from a given individual’s fecal sample [according to IMGG sample attributes described previously (Jin *et al.* 2023)]. For each type of library generation and marker gene evaluated, the percent of genome-level diversity captured at each cutoff was calculated as:

(1) $$\mathrm{Genome}\;\mathrm{capture}\;\mathrm{rate}=\frac{\mathrm{Number}\;\mathrm{of}\;\mathrm{sequence}\;\mathrm{groups}\;\mathrm{generated}\;\mathrm{by}\;\mathrm{isoLIB}}{\mathrm{Number}\;\mathrm{of}\;\mathrm{unique}\;\mathrm{input}\;\mathrm{genomes}}$$

whereas the percent of species diversity captured was calculated as:

(2) $$\mathrm{Species}\;\mathrm{capture}\;\mathrm{rate}=\frac{\mathrm{Number}\;\mathrm{of}\;\mathrm{unique}\;\mathrm{species}\;\mathrm{represented}\;\mathrm{by}\;\mathrm{isoLIB}\;\mathrm{sequence}\;\mathrm{groups}\;}{\mathrm{Number}\;\mathrm{of}\;\mathrm{unique}\;\mathrm{species}\;\mathrm{represented}\;\mathrm{by}\;\mathrm{input}\;\mathrm{genomes}}$$

Accordingly, these ratios provide insight into how marker gene selection impacts the absolute number of strains that can be differentiated relative to the taxonomic coverage obtainable at each sequence similarity cutoff. As a point of reference for future culture collection initiatives, we also assessed the entire IMGG dataset [12 391 genomes, including those of varying completeness and quality (Jin *et al.* 2023)] as well as a larger dataset from the non-industrialized diverse microbiomes of Hadza hunter-gather populations [48 475 genomes from ultra-deep sequencing (Carter *et al.* 2023)] to comparatively evaluate how intraspecies genome diversity differs at the population and individual levels. The bash scripts used in the meta-analysis are available at: https://bdaisley.github.io/isolateR/benchmark/figure5_sh_script.html. The R scripts for visualizing meta-analysis results are available at: https://bdaisley.github.io/isolateR/benchmark/figure5_r_script.html.

## 3 Results

**Figure 2.**
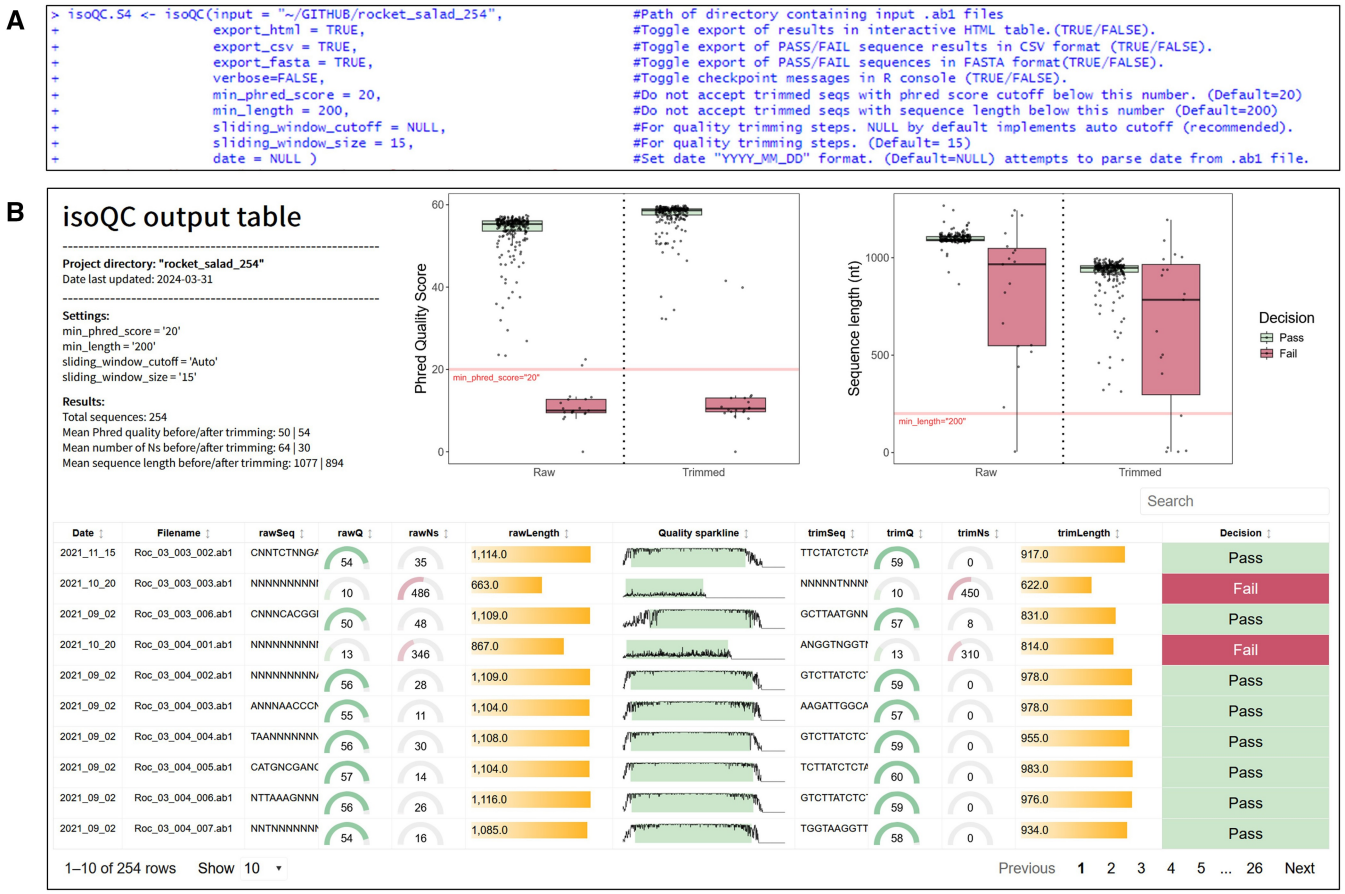
isoQC function overview. (A) Input parameters. (B) Interactive HTML output table of results. The “Quality Sparkline” column shows the Phred quality over the entire sequence with the highlighted area representing the trimmed region of the sequence.

To illustrate the usage of isolateR (Fig. 2), we analyze data from 254 bacterial isolates (16S rRNA gene Sanger sequence files downloaded from: https://dataverse.unimi.it/dataverse/vegmicroecol), which were originally derived from either conventional or organic ready-to-eat rocket salads found in local supermarkets across Milan, Italy (Mantegazza *et al.* 2023). This is a subset of the example data included in the isolateR package and the entire analysis including data import, quality trimming of sequences, taxonomic classification, and creation of the bacterial strain library can be completed by running three lines of R code. We discuss the execution and results of this workflow in more detail below.

### 3.1 Step 1: quality processing of Sanger sequences

Upon specifying the location of the directory containing the Sanger sequence files, the *isoQC* function can be run with default settings to automatically perform basecalling and adaptive quality trimming for all sequences in a single run (Fig. 2A). In the interactive HTML output (Fig. 2B), users can inspect descriptive statistics of the input sequences before and after trimming. The “Quality Sparkline” column further highlights the exact trimming region for each sequence, and the “Decision” column indicates whether a sequence should be discarded or not based on specified quality thresholds.

In this example, we see that the adaptive trimming approach in isolateR leads to a substantial improvement in the overall quality of sequence data. The mean length of input sequences is approximately 1077 bp before trimming and 894 bp after trimming. In tandem, there is a substantial reduction in the number of ambiguous bases decreasing from 64 to 30 with Phred quality scores increasing from an initial mean of approximately 50 to over 54 post-trimming (Fig. 2B).

### 3.2 Step 2: taxonomic classification of sequences

**Figure 3.**
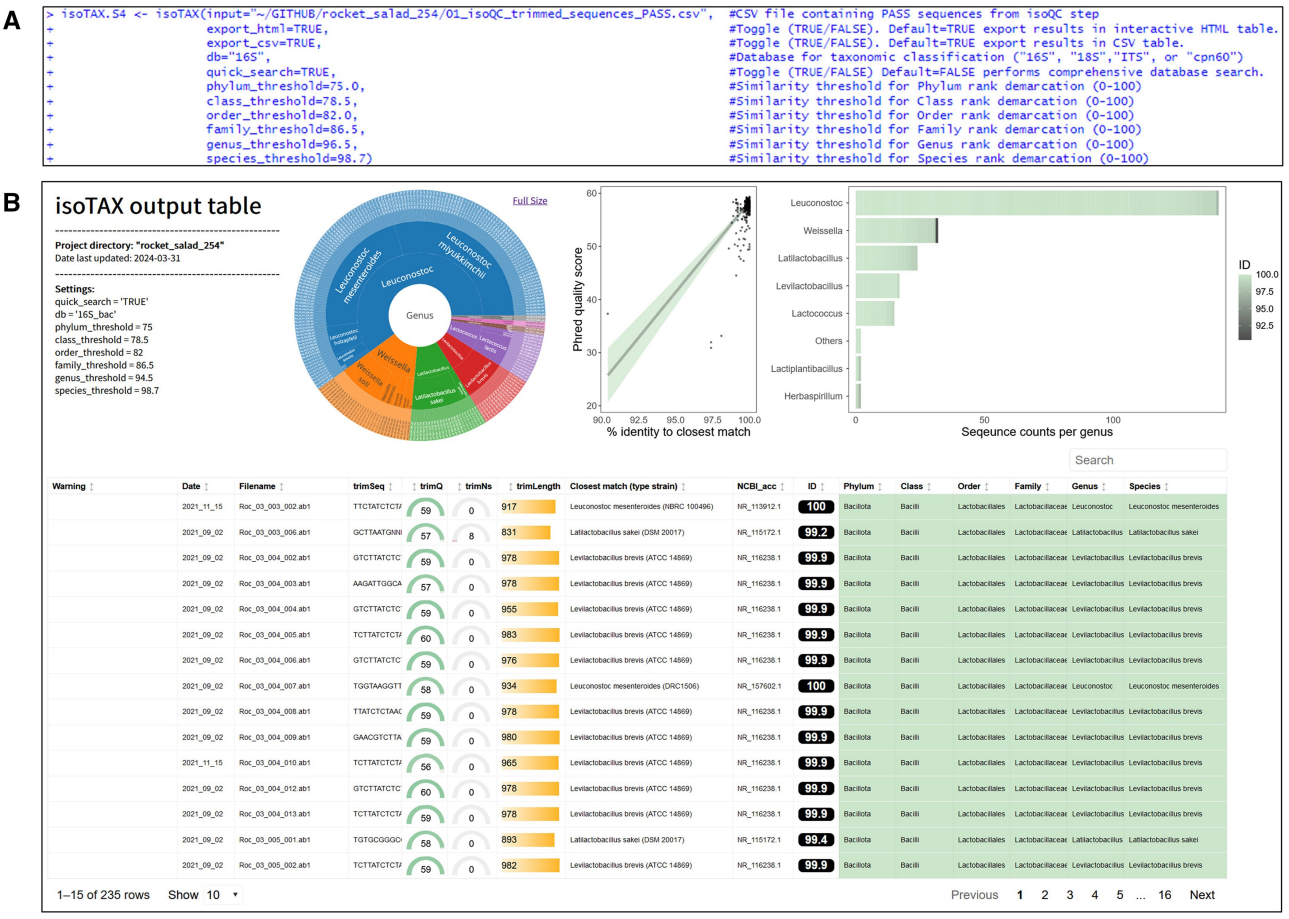
isoTAX function overview. (A) Input parameters. (B) Interactive HTML output table of results. “ID” column represents the pairwise global alignment sequence similarity of query sequences compared to the closest matching reference sequence from type strain database of interest. Taxonomic rank columns on right are highlighted based on the phylum- to species-level demarcation thresholds specified. Highlighted cells suggest confident taxonomic identity at each of the specified ranks, whereas unhighlighted cells are indicative of potentially novel taxa.

For common marker gene sequences such as 16S rRNA, taxonomic classification can be performed by running the *isoTAX* function with default settings. In the interactive HTML output (Fig. 3), the closest taxonomic matches and respective pairwise sequence identities are shown for each of the sequence representatives. Additionally, according to established taxonomic demarcation thresholds (Yarza *et al.* 2014), each of the taxonomic rank columns (phylum, class, order, family, genus, species) are shaded green based on whether sequence identities are supported at each level.

In the current example, we see several samples (Roc_04_005_005, Roc_04_006_001, Roc_04_006_005, Roc_04_006_012, and Roc_04_009_001) that have 100% sequence identities matching to *Lactococcus lactis* NCDO 604, and thus may represent clonal isolates. In contrast, there are two samples (Roc_03_013_001 and Roc_03_013_006) with sequence identities below the species demarcation threshold of 98.7%, suggesting these isolates may represent novel species found in association with rocket salad; these differences are visually reflected by an absence of green shading in the taxonomic species rank columns to help users rapidly identify potentially novel taxa of interest.

As an additional quality check, Phred quality scores for each sample are plotted against their respective sequence identities to the closest matching type strains, allowing outliers to be visually inspected (four samples in the current example with Phred scores less than 40) and removed from downstream analysis if applicable. Furthermore, a stacked bar plot and interactive sunburst chart are generated which highlight the most commonly detected taxa and their compositionality in the sample set, respectively (Fig. 3B).

### 3.3 Step 3: strain library creation

**Figure 4.**
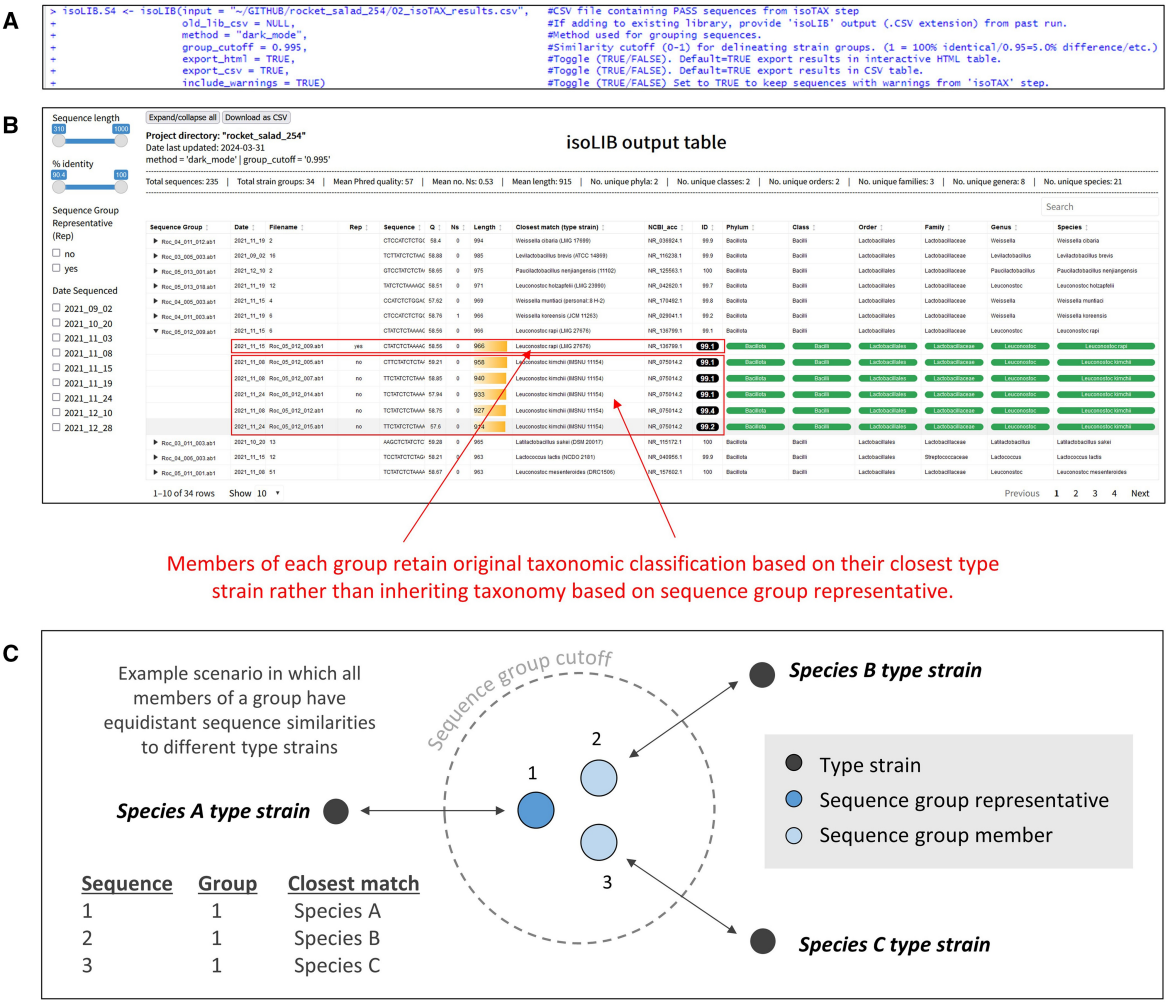
isoLIB function overview. (A) Input parameters. (B) Interactive HTML output table of results. Strain groups are based on sequence similarity at the specified threshold of interest. Members of each unique group are listed under the “filename” column and the sequence representatives (rep) are indicated in the “Rep” column. Taxonomic rank columns on right are highlighted based on the phylum- to species-level demarcation thresholds specified. Highlighted cells suggest confident taxonomic identity at each of the specified ranks, whereas unhighlighted cells are indicative of potentially novel taxa based on thresholds set. Interactive widgets allow text-based searching as well as filtering of the strain library based on date, sequence length, pairwise identity, and other metadata. (C) Schematic overview illustrating how sequences in the same group can be very closely related based on sequence identity yet have different taxonomic classifications based on closest matching type strain sequence identities.

The final step involves generating a strain library using the *isoLIB* function (Fig. 4). This function enables the dereplication of clonal or closely related isolates based on customizable sequence similarity thresholds and offers flexible creation of libraries catering to specific research needs and the desired level of intraspecies or interspecies diversity. Interactive widgets in the HTML output document allow text-based searching as well as filtering of the strain library based on date, sequence length, pairwise identity, and other metadata.

In the example rocket salad culture collection (Fig. 4A and B), we see that the 235 sequences passing quality steps are represented by a total of 21 unique species and can be dereplicated to 34 strain groups based on the default grouping method (“dark_mode”) and cutoff (0.995 = 99.5% sequence similarity). Each of the sequence group rows can be expanded *via* the dropdown menu to further inspect each of the samples within a given grouping. The top five groups by size are represented by *Leuconostoc miyukkimchii* (67), *Leuconostoc mesenteroides* (51), *Weissella soli* (17), *Levilactobacillus brevis* (16), and *Latilactobacillus sakei* (13).

Notably, de-replicating sequences at a given cutoff as in the “dark_mode” method often leads to the grouping of organisms with disparate taxonomies, a consequence of their equidistant sequence identities to closely matched type strains (Fig. 4C). This behavior aligns with an ecological species concept (Shapiro and Polz 2015), aiming to optimally organize distinct biological units according to their relevance in a specific habitat. For instance, in the rocket salad dataset, one of the samples, Roc_05_012_009, showing 99.1% identity to *Leuconostoc rapi*, groups with other samples (Roc_05_012_005, Roc_05_012_007, Roc_05_012_014, Roc_05_012_012, and Roc_05_012_015) identified closer to *Leuconostoc kimchii*, with identities ranging from 99.1% to 99.4% (Fig. 4C). Such grouping suggests these samples might represent a novel species or other biological unit meriting further exploration. Despite the apparent taxonomic diversity, the close relationship between these samples and their collective distinction from type strains nonetheless underscore the method's efficacy in de-replicating clonal or highly similar isolates. Transitioning from the ecological considerations enabled by “dark_mode”, the *isoLIB* “closest_species” method addresses a different set of research objectives by enabling the creation of libraries where all members of a specific group are uniformly assigned the same taxonomic classification. This capability illustrates the platform's versatility in supporting a broad spectrum of study designs.

Emergent patterns of strain distribution and correlations with metadata attributes like sample source or collection date may further support hypothesis generation about ecological roles and interactions of specific taxa. Additionally, comparative analysis of strain libraries may reveal differences in strain diversity and abundance across habitats or conditions, providing valuable information for diagnostic or therapeutic applications.

### 3.4 Benchmark analysis and practical considerations

**Figure 5.**
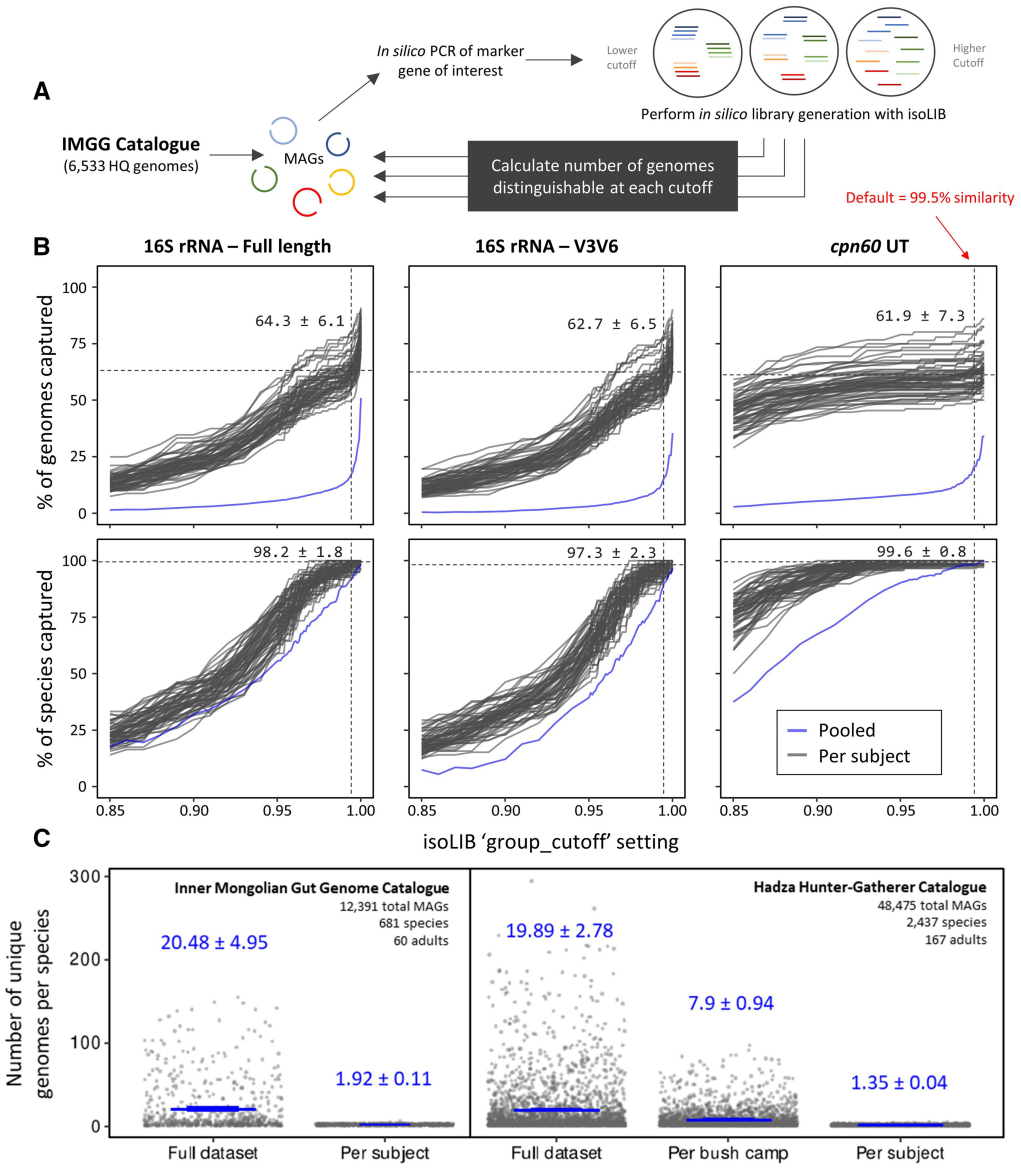
*In silico* benchmark of generating isolate libraries from the human gut. The *isoLIB* function aims to improve isolation workflow efficiency by grouping clonal or very closely related strains based on pairwise sequence identity of a given marker gene. Here, we assess a set of high-quality bacterial genomes from the Inner Mongolian Gut Genome (IMGG) catalogue to practically evaluate how the usage of different marker genes and *isoLIB* “group_cutoff” settings may impact diversity of culture collections from the human gut. (A) Schematic overview of methodology. A subset of 6533 high-quality (HQ) bacterial genomes from the IMGG catalogue, which contained both 16S rRNA and *cpn60* marker genes, were extracted *via in silico* PCR, and then iteratively grouped with *isoLIB* to simulate the dereplication of closely related strains based on sequence identity. (B) Results showing the percent of unique genomes relative to unique species expected to be captured at each cutoff when library generation is based on full-length 16S rRNA, V3–V6 regions 16S rRNA, or *cpn60* universal target (UT) marker genes. In every instance, capturing genome-level variation is enhanced by analyzing the metagenomes of individuals separately—underscoring the value of creating culture collections from individual niches as opposed to pooled samples. (C) Point of reference overview of the entire IMGG and Hadza Hunter–Gather catalogues showing intraspecies genome variability of the human gut at different scales.

While the concept of a strain and the interpretation of species-level diversity of strains within microbiomes have been subject to debate (Van Rossum *et al.* 2020), adopting the perspective that any microbe with a distinct genome constitutes a strain allows for a broader and more inclusive understanding of microbial diversity. Here, we establish a real-case benchmark on how different *isoLIB* settings are expected to impact cataloging of strain-level diversity from the human gut by conducting an evaluation on 6533 HQ metagenomic assembled genomes (MAGs) obtained from 60 individuals from Inner Mongolia (Jin *et al.* 2023). Focusing on the typical scenario of using either full-length 16S rRNA, the V3–V6 regions 16S Rrna, or *cpn60* marker genes to identify bacterial isolates, we find that 3335, 2323, and 2224 MAGs, respectively, are distinguishable by at least a single nucleotide polymorphism or more compared to others within the pooled set of 6533 (Fig. 5A and B). This means that by applying a “group_cutoff = 1” for identical sequence dereplication in the *isoLIB* process, we could potentially capture about 34–51% of the overall strain diversity depending on the marker gene chosen. In contrast, using a “group_cutoff = 0.987”—reflecting the general species-level demarcation threshold for full-length 16S rRNA (Yarza *et al.* 2014)—results in capturing less than 15% of the strain diversity. However, this subset encompasses over 86% of the unique species found across the 60 individuals in the IMGG catalogue. At the same cutoff, the *cpn60* UT [previously shown to have greater sensitivity in demarcating phenotypically distinct sub-species and ecotypes (Vermette *et al.* 2010, Katyal *et al.* 2016)] captures a similar number of strains, which represent over 97% of species-level diversity. Together, these results align with other large-scale studies (Huttenhower *et al.* 2012, Gilbert *et al.* 2018, Pasolli *et al.* 2019), underscoring that strain-level variation within gut microbiomes is extensive at the population level and further highlighting the critical importance of choosing appropriate genetic markers and case-specific dereplication thresholds to accurately capture microbial diversity in isolation workflows.

Notably, strain-level resolution is greatly enhanced if we separately consider each of the gut microbiomes from the 60 Inner Mongolians as distinct habitats. For example, nearly double the number of MAGs (64–81% depending on marker gene) are distinguishable per individual at “group_cutoff = 1.0”, and at the default setting (“group_cutoff = 0.995”) all marker genes show high consistency in capturing approximately 61–64% of strain diversity and 97–99% of species-level diversity. Moreover, the rate at which decreasing the “group_cutoff” setting of *isoLIB* reduces the theoretical rate of strain capture is less rapid than when considering all strains pooled together across individuals (Fig. 5B). This phenomenon may be explained by the observation that conspecific strains sharing the same ecological niche frequently exhibit significant genomic divergence, which is attributable to variations in complementary accessory gene combinations underlying their co-existence (Hu *et al.* 2022). Exemplifying this point in relation to diseased host states, Wang *et al.* (2021) recently showed that over 70% of adherent-invasive *Escherichia coli* strains from inflammatory bowel disease patients could be efficiently tracked and quantified based on 16S rRNA identity—despite the fact that *E. coli* strains across individuals and environments are exceptionally challenging to differentiate based on any single gene marker (Hu *et al.* 2022). Thus, in cases of isolating microbes from habitats with high strain-level diversity (such as the human gut), it is advisable to generate libraries on a per individual host or habitat basis, to gain the greatest resolution in strain diversity.

As a point of reference further illustrating the effect of cataloging microbes from diverse habitats, we perform a secondary analysis on the non-industrialized microbiomes of Hadza hunter-gathers (Carter *et al.* 2023). Similar to the IMGG dataset, we show there are approximately 20 distinct MAGs detected per species unit across the 167 Hadza individuals, whereas each individual’s microbiome is estimated to possess less than two distinct MAGs (strains) per species unit (Fig. 5C). Hadza individuals living in close proximity within specific “bush camps” are known to share microbes with one another extensively (Carter *et al.* 2023). Reflecting this unique lifestyle, we show that when individual microbiomes are pooled based on “bush camp”, the number of MAGs per species unit increases only approximately ∼7, indicating a gradient zone where interindividual strain variability is less than expected. Nonetheless, both the IMGG and Hadza datasets support generating culture collections on a per-individual basis to capture the greatest strain-level diversity.

An important caveat is that these statistics are based on the assumption that all strains are culturable and have equal chances of being picked during isolation workflows. Since this is rarely the case, there are often tradeoffs when it comes to capturing the continuum of conspecific strain diversity relative to the overall taxonomic breadth attainable with consideration for throughput during isolation procedures. The default isoLIB parameter (“group_cutoff = 0.995”) aims to strike a balanced compromise in terms of functionally grouping closely related strain variants, preventing clonal isolates, and ultimately expediting isolation workflow efficiency. Based on our analysis of the IMGG catalogue and the three primer sets tested, this default setting is estimated to capture 97–99% of bacterial species and approximately 61–64% of strain diversity when isolations are performed at the individual level. However, different taxonomic markers may be better or worse suited for delineating strains from a given habitat, and these estimates may not be generalizable to other domains of microbial life such as fungi and archaea, which have varying criteria for classifying species (Abarenkov *et al.* 2024). Thus, users may adjust this parameter higher or lower depending on desired study goals. These findings together serve as a useful benchmark for future culture collection endeavors and as a reminder of the inherent limitations of marker genes for delineating strain-level genetic diversity.

### 3.5 Comparisons to existing software

Table 1 details the similarities and differences between isolateR and other relevant software including sangeranalyserR (Chao *et al.* 2021), CAP3 (Huang and Madan 1999), DECIPHER (Wright 2016), Geneious (Kearse *et al.* 2012), MEGA7 (Kumar *et al.* 2016), SeqTrace (Stucky 2012), TraceTrack (Brazdilova *et al.* 2023), and LPSN API (Meier-Kolthoff *et al.* 2022).

**Table 1.** Comparisons between isolateR and existing software.

Features	isolateR	Geneious	LPSN-API	sangeranalyseR	DECIPHER	CAP3	MEGA7	SeqTrace
Batch processing of Sanger files	X	X		X		X	X	X
Automated quality trimming of DNA sequences	X	X		X	X	X	X	X
Automated contig assembly	X	X		X	X	X	X	X
Phylogenetic tree output	X	X	X		X		X	
Taxonomic classification	X		X		X			
Dynamic re-assignment of taxonomy based on International Codes of Nomenclature	X		X					
Generation of sequence libraries	X							

Many software options provide trace visualization and sequence quality trimming, either manually or automatically (Stucky 2012, Chao *et al.* 2021, Brazdilova *et al.* 2023), but lack in taxonomic classification features, which are typically offered by a mutually exclusive set of specialized tools (Wright 2016). isolateR takes a significant step forward by automating taxonomic classification directly from Sanger sequencing input files, while also allowing dynamic re-assignment of taxonomy to support the ever-evolving nature of hierarchical ranking systems in accordance with the rigorous standards of International Codes of Nomenclature (Meier-Kolthoff *et al.* 2022). Beyond processing and annotation features, isolateR is specifically tailored for generating sequence libraries that are instrumental in identifying novel taxa and in the efficient creation of large-scale microbial culture collections.

Ultimately, what sets isolateR apart is its ability to bridge the gap between functionalities of multiple bioinformatic tools, providing a comprehensive solution that addresses the varied needs of microbial genomics research. This integration simplifies microbial isolation workflows and unites the path from sequence acquisition to taxonomic classification and culture collection creation. Accordingly, these features position isolateR as a valuable tool for researchers focused on expanding our understanding of microbial diversity and streamlining the management of microbial resources.

## 4 Discussion

The entire isolateR workflow, from data import to the creation of the strain library can be completed efficiently using just three lines of R code. The processing time for the entire example dataset was under 15 min, demonstrating the software's capability to handle moderately large datasets with speed and accuracy within a standard laptop computing environment. In summary, isolateR is a robust and user-friendly tool supporting the high-throughput analysis of Sanger-based marker sequence data, comprehensive taxonomic classification, and straightforward creation of microbial strain libraries.

## Data Availability

The source code and data are available at https://github.com/bdaisley/isolateR.
